# A Preliminary Study on the Effect and Mechanism of Antarctic Krill Peptides AKP on Alleviating UV-Induced Skin Photoaging by Regulating Estrogen Signaling Pathway

**DOI:** 10.3390/md24100342

**Published:** 2026-09-29

**Authors:** Lan Wang, Wen Weng, Wanxiu Cao, Fen Du, Xiaomei Feng, Xueyuan Fu, Changwei Wang, Bafang Li, Yan Xing, Qingjuan Tang, Chuyi Liu

**Affiliations:** 1 Marine Biomedical Research Institute of Qingdao, Ocean University of China, Qingdao 266100, China; wanglan2608@163.com (L.W.); wwen1192@163.com (W.W.);; 2 State Key Laboratory of Marine Food Processing & Safety Control, College of Food Science and Engineering, Ocean University of China, Qingdao 266003, China; 3 Qingdao Marine Biomedical Research Institute Co., Ltd., Qingdao 266100, China

**Keywords:** skin photoaging, Antarctic krill peptides, bioactive peptides, estrogen signaling pathway, UV induction

## Abstract

Chronic ultraviolet (UV) exposure is a primary driver of skin photoaging, yet safe and effective natural interventions remain limited. This study investigated the anti-photoaging potential and mechanism of Antarctic krill peptides (AKP), a low-molecular-weight oligopeptide mixture (Mw ~331 Da, with 82.82% <500 Da) prepared by enzymatic hydrolysis and membrane fractionation. AKP exhibited excellent skin permeability in vitro. In UV-irradiated human dermal fibroblasts (HDF), AKP alleviated oxidative damage, restored superoxide dismutase activity, reduced malondialdehyde content, and promoted collagen I and XVII secretion, and inhibited matrix metalloproteinase-1 (MMP-1) expression. In a chronic UV-induced mouse photoaging model, topical AKP ameliorated epidermal thickening, dermal collagen loss, and macroscopic wrinkle formation. Skin proteomics revealed that AKP upregulated estrogen receptor Esr1 and androgen receptor Ar, activating the estrogen signaling pathway, which in turn corrected aberrant keratin (Krt5/14/19) and cell-cycle protein (Cdkn1a/Ccna2) expression. Immunohistochemical validation confirmed that AKP inhibited MMP-1/9 overexpression, elevated tissue inhibitor of metalloproteinase-1 (TIMP-1), and increased deposition of collagens I, III, and XVII, thereby restoring extracellular matrix homeostasis. Collectively, these findings demonstrate that AKP combats skin photoaging through estrogen-mediated regulation of matrix degradation and repair. This work fills a mechanistic gap for Antarctic krill-derived peptides and supports their development as natural marine-based anti-aging agents.

## 1. Introduction

Skin is the body’s largest barrier organ, composed of epidermis, dermis, and subcutaneous tissue. It protects internal structures from external environmental factors, but is extremely vulnerable to damage [1,2]. Skin aging is a complex physiological process, which can be divided into endogenous and exogenous skin aging [3]. The latter is driven by external factors, among which ultraviolet (UV) radiation is the predominant contributor, a process termed photoaging [4]. Photoaged skin is histologically characterized by fibroblast depletion, inflammatory infiltration, degradation of collagen and elastin, clinically manifesting as dryness, thickening, loss of elasticity, and pigmentary unevenness [5,6,7].

The pathogenesis of UV-induced skin aging involves multiple interrelated mechanisms, including oxidative stress imbalance, DNA damage accumulation, chronic inflammatory, abnormal extracellular matrix (ECM) degradation, apoptosis, and immunosuppression [8,9,10]. Current therapeutic strategies, such as retinoids, topical retinol, antioxidants, and laser therapy, offer partial benefits in reducing wrinkles and pigmentation [11,12], but prolonged use often leads to adverse effects like irritation, dermatitis, and desquamation [13,14]. Therefore, there is growing interest in identifying natural, highly efficacious, and low-toxicity anti-photoaging agents.

Marine-derived bioactive peptides have emerged as promising candidates due to their abundant sources, adaptability to extreme environments, and diverse biological activities [15,16]. For instance, gelatin polypeptides from Pacific cod protect against UV-induced photodamage by suppressing inflammation and regulating the TGF-β/Smad pathway [17], while peptides from *Pinctada martensii* reduce MMP-1 and MMP-3 expression and enhance elastase inhibitory activity in HaCaT cells to achieve an anti-photoaging effect [18]. However, the mechanisms underlying many active marine peptides remain insufficiently explored, underscoring the need for further mechanistic elucidation.

Antarctic krill (*Euphausia superba*), with its vast biomass and rich nutritional profile, represents a valuable source of high-quality protein containing all essential amino acids [19,20,21]. Compared with intact proteins, Antarctic krill peptides (AKP) are small in molecular weight, easy to absorb, and exhibit rich biological functions due to the diversity of amino acid sequences and spatial structures. Studies have shown that Antarctic krill peptides have antioxidant [22], anti-inflammatory [23], immune regulation [24], anti-fatigue [25] and other active effects. Notably, Antarctic krill is also rich in lipophilic compounds such as astaxanthin, which may facilitate skin penetration and interaction with membrane receptors, partly underpinning the favorable transdermal properties observed in this study [26]. However, while antioxidant activities of AKP have been reported, its effects on key skin structural proteins and basement membrane repair remain unexplored.

In this study, we prepared low molecular weight Antarctic krill peptides (AKP) by membrane separation, characterized its molecular distribution and transdermal permeability, and evaluated its anti-photoaging efficacy in both cellular and animal models. Furthermore, we employed skin proteomics to identify key pathways, followed by immunohistochemical validation of critical proteins. Our findings provide novel mechanistic insights into AKP-mediated photoaging protection and offer a theoretical foundation for developing marine-derived natural anti-aging skincare products.

## 2. Results

### 2.1. Preparation and Characterization of AKP

**Figure 1:**
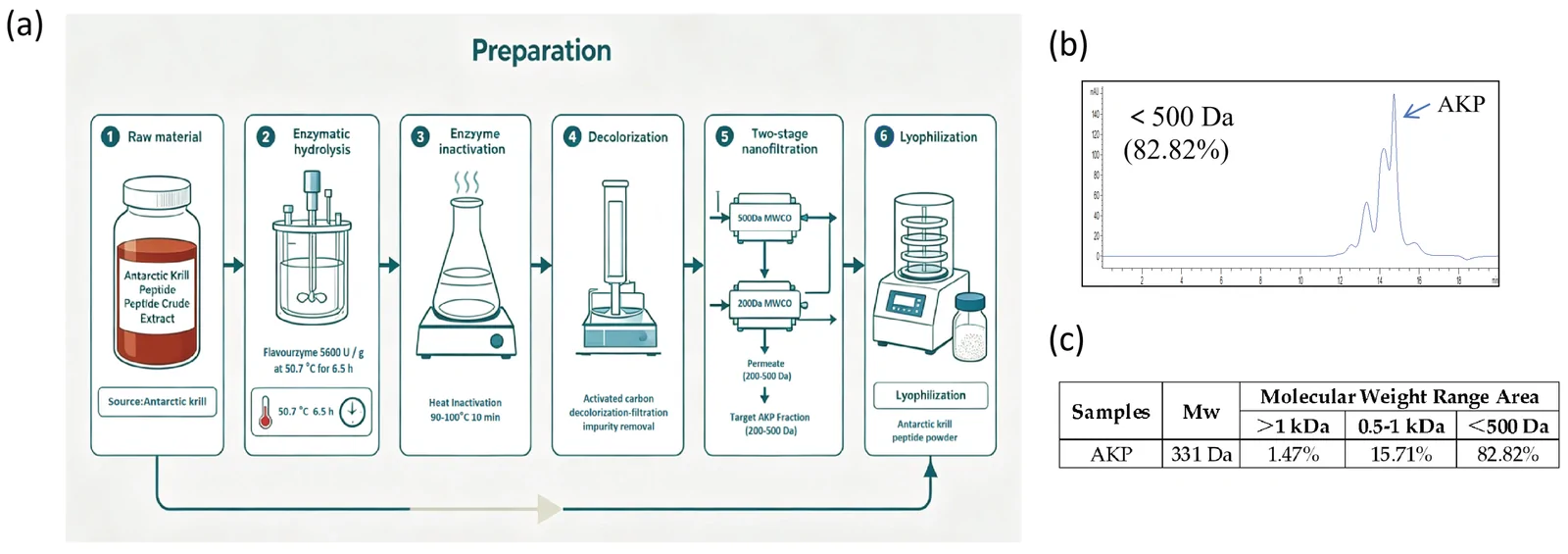
Preparation and molecular weight determination of AKP. (**a**) A schematic diagram of the workflow for preparing AKP from defatted Antarctic krill meal by enzymatic hydrolysis, membrane separation and freeze-drying; (**b**) HPLC-SEC chromatogram of AKP; (**c**) the molecular weight distribution of AKP.

Antarctic krill peptide (AKP) is a low molecular weight peptide mixture prepared by enzymatic hydrolysis and organic membrane fractionation using low-fat and high-protein defatted Antarctic krill powder as raw material. The specific process includes enzymatic hydrolysis, thermal inactivation, decolorization, using 500 Da, 200 Da molecular weight filter membrane to intercept, and collecting 200~500 Da components to be freeze-dried into powder for subsequent experiments. The preparation flow chart is shown in Figure 1a.

The molecular weight of AKP was determined by high performance liquid chromatography size exclusion chromatography (HPLC-SEC) after the molecular weight of AKP was prepared by molecular weight fractionation (Figure S1). As shown in Figure 1b, the peak of liquid chromatography shifted back regularly, the peak shape gradually narrowed, and the molecular weight distribution tended to be uniform. Further analysis and calculation of the HPLC diagram showed that the molecular weight distribution was shown in Figure 1c. The average molecular weight of AKP was 331 Da, and the proportion of peptides below 500 Da was 82.82%, indicating that the classification was sufficient. The peptide sequence of AKP was identified by LC-MS/MS technology, and a total of 1685 peptides were identified. Sorted in descending order of expression abundance, the information of the first 15 peptides is shown in Table 1. The results showed that the 15 peptides with the highest abundance in AKP were mainly tripeptides (14 in total), only one was tetrapeptide (VPGV), and the molecular weight was concentrated in 285–456 Da, all of which belonged to the range of small molecular peptides. The abundance of peptides below tetrapeptides in AKP was counted, and tripeptides accounted for 62.16% and tetrapeptides accounted for 31.27%, which was consistent with the previous HPLC results. This result can provide a molecular basis for subsequent in vivo efficacy verification and mechanism research.

### 2.2. In Vitro Transdermal Absorption Properties of AKP

**Figure 2:**
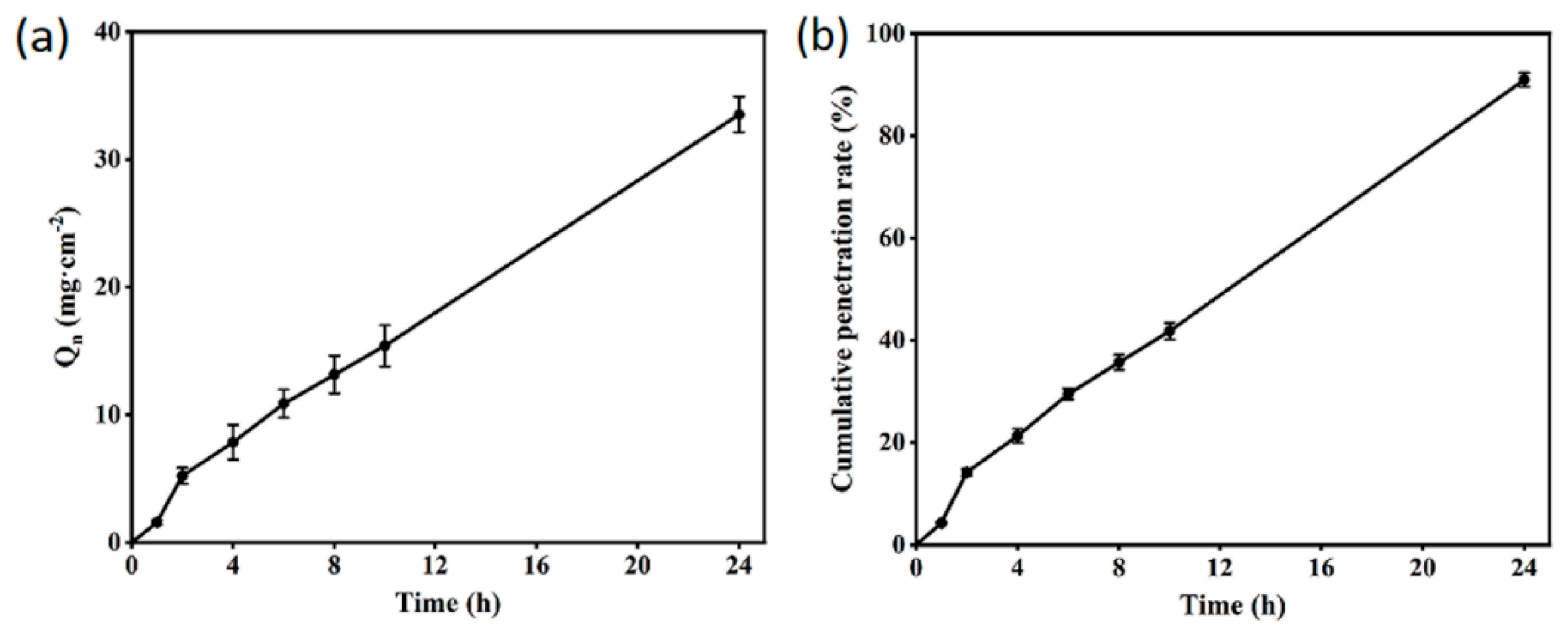
Changes in the cumulative transmittance of AKP per unit area. (**a**) Cumulative transmittance per unit area; (**b**) cumulative transmittance. Results were expressed as mean ± SD (*n* = 6).

Transdermal absorption is a prerequisite for the topical peptide to exert a local anti-photoaging effect on the skin, which can be used to verify the feasibility of AKP for external delivery. Figure 2 shows the cumulative transmittance per unit area (a) and cumulative transmittance curve (b) of AKP within 24 h. The results showed that AKP had excellent transdermal absorption performance, and its cumulative permeation amount per unit area (*Q_n_*) and cumulative permeation rate continued to increase with time. At 24 h, *Q_n_* was 33.51 ± 0.51 mg·cm^−2^, and the cumulative permeation rate was 90.94%, indicating that AKP had excellent skin penetration ability and continuous transdermal delivery characteristics during the observation period.

### 2.3. Effect of AKP on the Survival Rate of Photoaged HDF Cells

**Figure 3:**
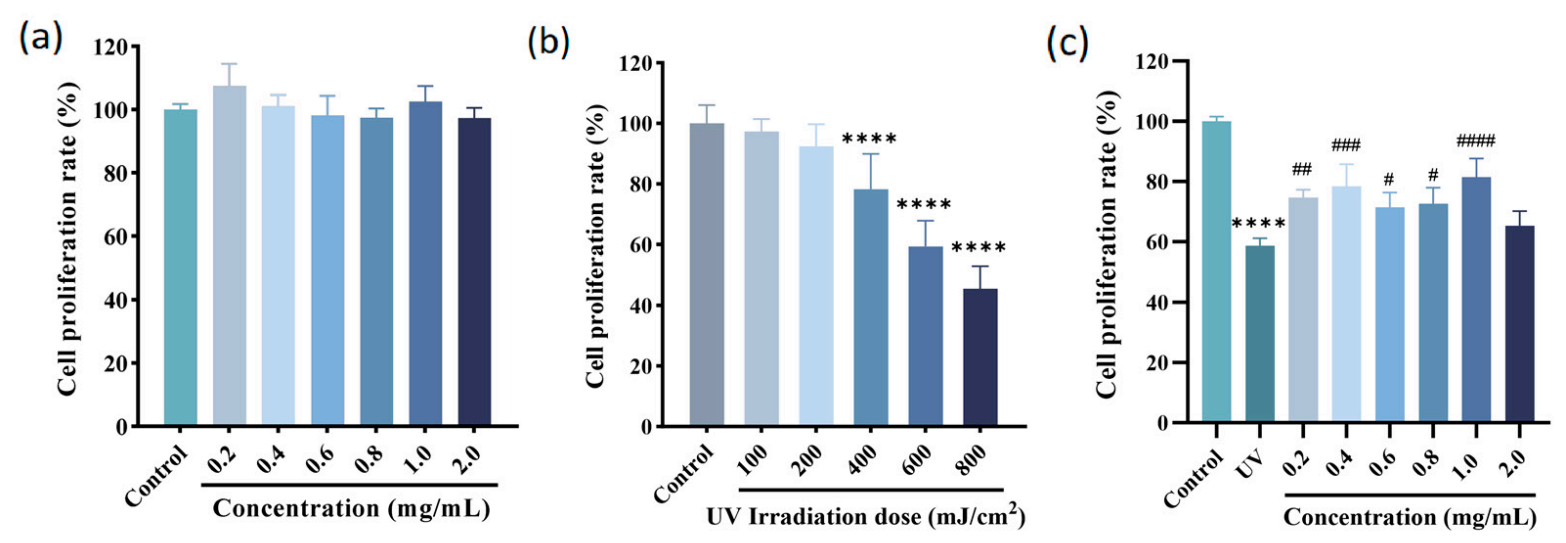
Effect of AKP on the survival rate of photoaged HDF cells. (**a**) Cytotoxicity of AKP on HDF cells; (**b**) the effect of different UV irradiation doses on the proliferation rate of HDF cells; (**c**) the effect of AKP on the proliferation rate of HDF cells after UV irradiation. Results were expressed as mean ± SD (*n* = 6). **** *p* < 0.0001 compared with Control group; ^#^ *p* < 0.05, ^##^ *p* < 0.01, ^###^ *p* < 0.001, ^####^ *p* < 0.0001 compared with UV group.

First, the cytotoxicity of AKP on HDF cells was detected by MTT assay, and the effects of different concentrations of AKP (0.2, 0.4, 0.6, 0.8, 1.0 and 2.0 mg/mL) on the survival rate of HDF cells were evaluated. As shown in Figure 3a, AKP has no cytotoxicity to HDF cells and has good biosafety. In order to construct the model successfully, HDF cells were exposed to different UV irradiation doses (100, 200, 400, 600, 800 mJ/cm^2^), and the appropriate irradiation dose was screened by the MTT method. As shown in Figure 3b, with the increase of UV irradiation dose, the density of HDF cells decreased in a dose-dependent manner. When the UV irradiation dose was 600 mJ/cm^2^, the cell survival rate was 59.39 ± 3.10%, close to 60%, so this dose was selected for subsequent cell experiments. Then, in order to explore the protective effect of AKP on photoaging HDF cells, AKP was added to HDF cells after UV irradiation to intervene. When the sample concentration was 0.4 and 1.0 mg/mL, AKP could significantly improve the cell viability of photoaging HDF cells (*p* < 0.001). Considering the cost-effectiveness and safety of long-term application, a lower concentration (0.4 mg/mL) was selected for all subsequent cell experiments.

### 2.4. Effect of AKP on the Production of Reactive Oxygen Species in Photoaged HDF Cells

**Figure 4:**
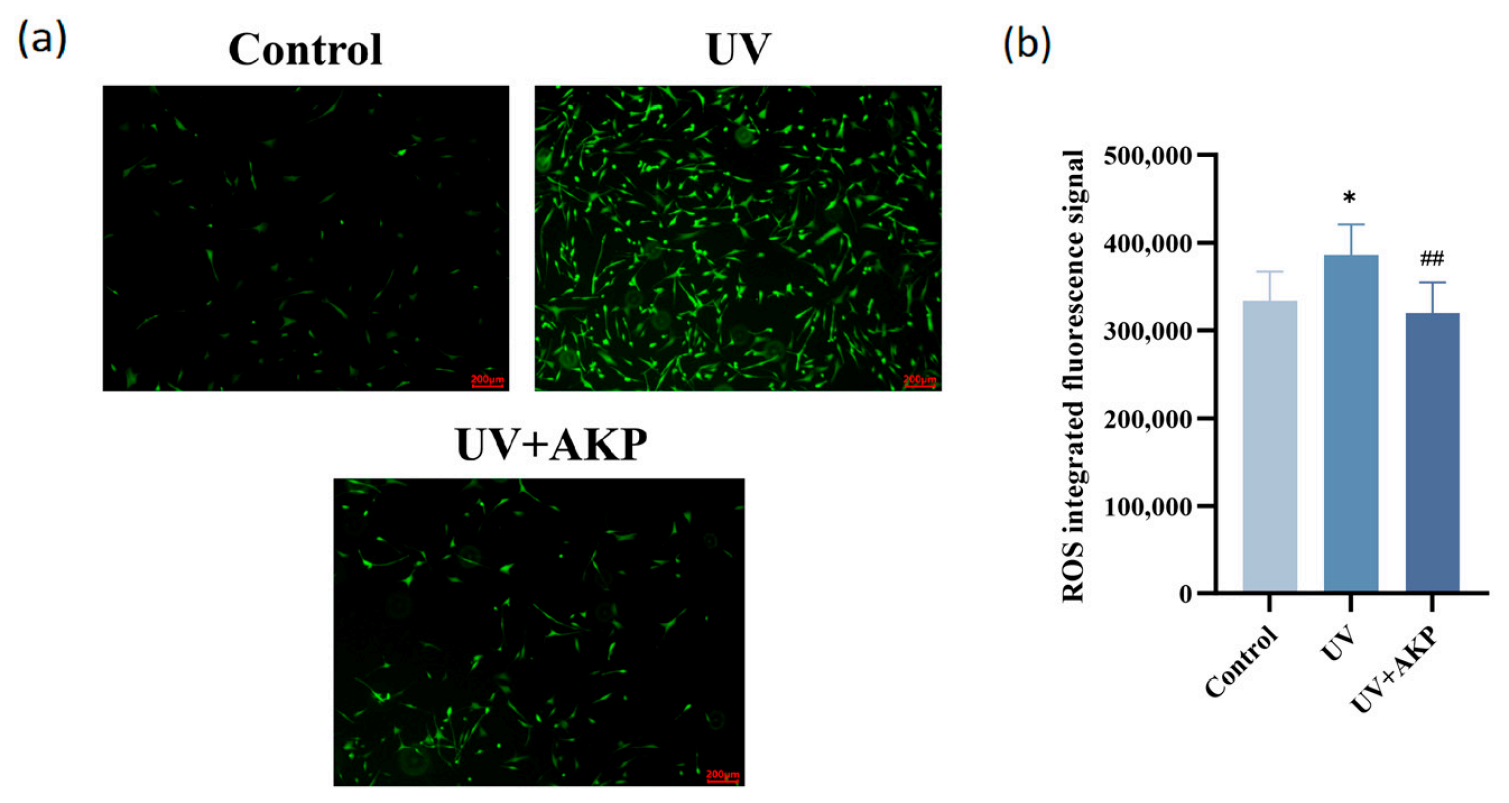
Effect of AKP on the production of reactive oxygen species in HDF cells after UV irradiation. (**a**) Reactive oxygen staining diagram (scale = 200 μm), green indicates ROS-positive cells within the cell; (**b**) reactive oxygen fluorescence signal. Results were expressed as mean ± SD (*n* = 6). * *p* < 0.05 compared with Control group; ^##^ *p* < 0.01 compared with UV group.

Ultraviolet irradiation can induce a large accumulation of reactive oxygen species (ROS) in cells. Excessive ROS can destroy the redox balance of cells and induce photoaging of cells. DCFH-DA fluorescent probe was used to detect the change of intracellular ROS level to evaluate the alleviation effect of AKP on oxidative stress in UV-induced photoaging HDF cells. The results are shown in Figure 4. Compared with the Control group, the number of ROS positive cells in the UV group was significantly increased, and the fluorescence intensity was significantly enhanced (*p* < 0.05). After AKP treatment for 24 h, the number of ROS-positive cells in the cells decreased, and the fluorescence intensity decreased significantly (*p* < 0.01), indicating that AKP treatment could effectively inhibit intracellular ROS production and alleviate UV-induced oxidative stress.

### 2.5. Effect of AKP on Antioxidant Activity of Photoaging HDF Cells

**Figure 5:**
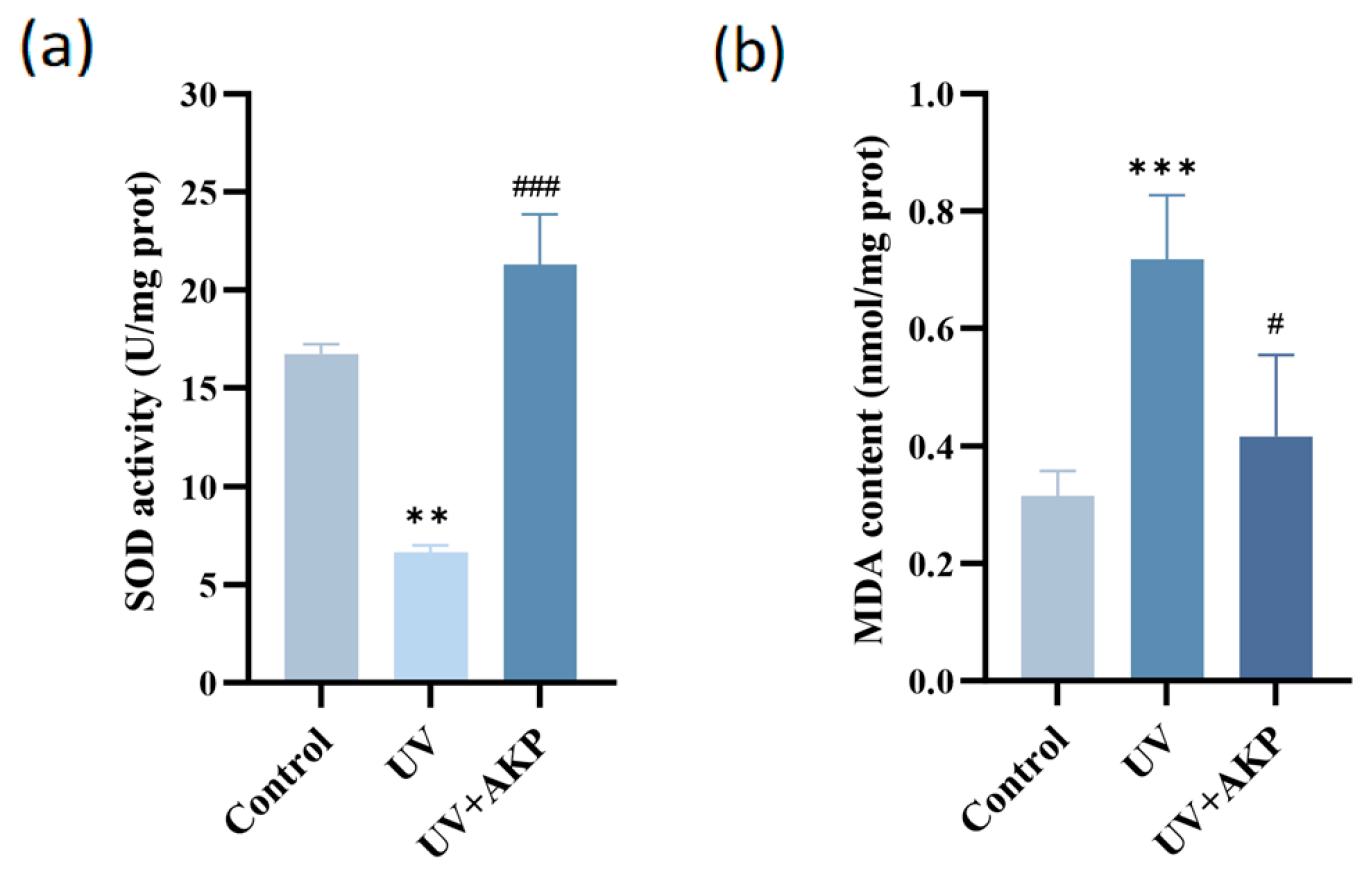
Effects of AKP on SOD activity and MDA content in HDF cells after UV irradiation. (**a**) SOD activity; (**b**) MDA content. Results were expressed as mean ± SD (*n* = 6). ** *p* < 0.01, *** *p* < 0.001 compared with Control group; ^#^ *p* < 0.05, ^###^ *p* < 0.001 compared with UV group.

Superoxide Dismutase (SOD) can maintain the redox balance of the body, and Malondialdehyde (MDA) can measure the degree of oxidative damage in the body. The combined detection functions of MDA content and SOD activity can fully reflect the severity of oxidative damage and the disorder of redox balance in the process of cell photoaging. As shown in Figure 5, compared with the Control group, the SOD activity in the UV group was significantly decreased (*p* < 0.01), and the MDA content was significantly increased (*p* < 0.001), suggesting that the intracellular redox homeostasis was unbalanced and in an oxidative stress state. After AKP treatment, the activity of SOD in photoaging HDF cells was significantly increased (*p* < 0.001), the content of MDA was significantly decreased (*p* < 0.05), and the oxidative stress state was significantly alleviated, which could improve UV-induced cell oxidative damage and delay cell photoaging.

### 2.6. Effect of AKP on Collagen Synthesis and Secretion of Photoaged HDF Cells

**Figure 6:**
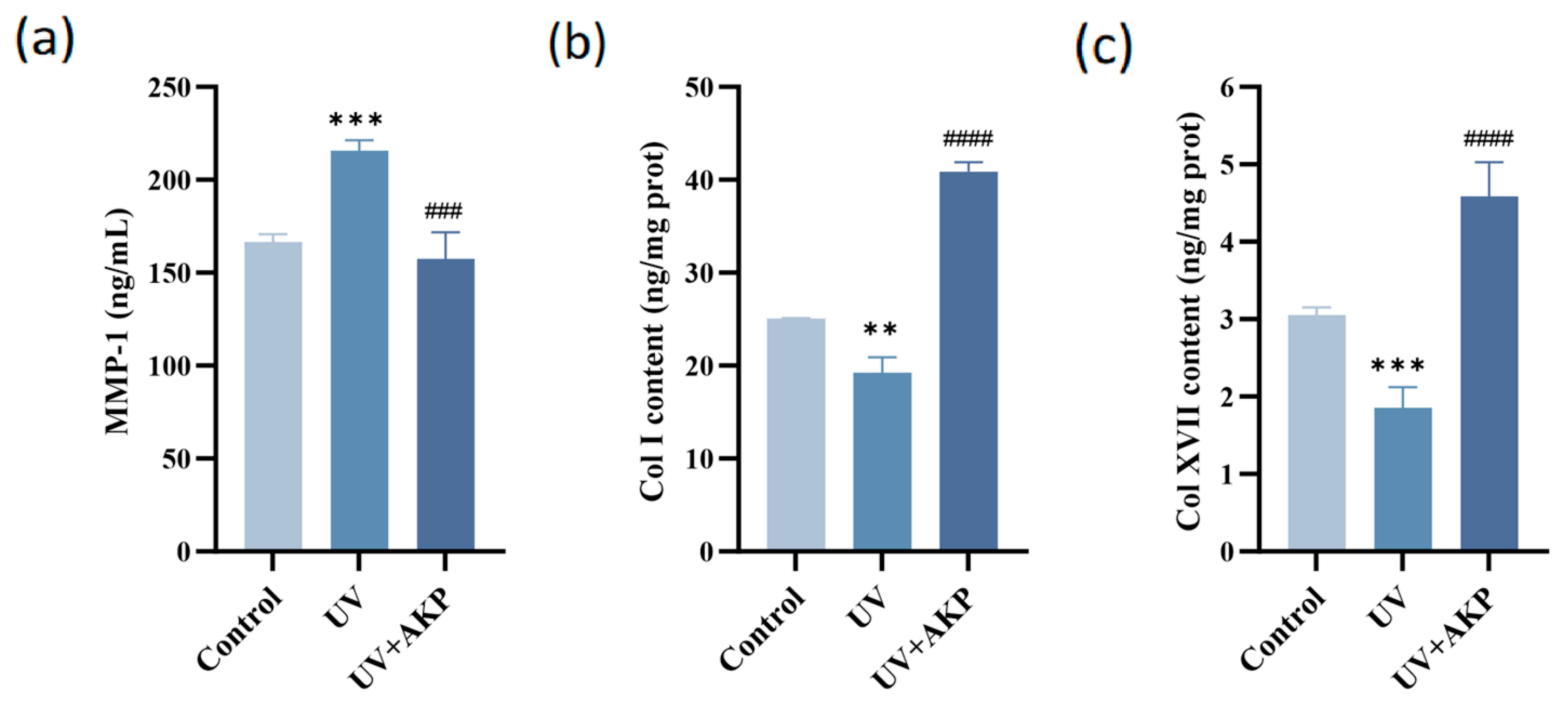
Effects of AKP on MMP-1 and collagen content in HDF cells after UV irradiation. (**a**) MMP-1 content; (**b**) collagen type I content; (**c**) XVII collagen content. Results were expressed as mean ± SD (*n* = 6). ** *p* < 0.01, *** *p* < 0.001 compared with Control group; ^###^ *p* < 0.001, ^####^ *p* < 0.0001 compared with UV group.

To investigate whether AKP can exert anti-photoaging activity by inhibiting collagen degradation and promoting collagen synthesis, the contents of MMP-1, type I collagen and XVII collagen in the cell culture medium of each group were measured. The results are shown in Figure 6. After UV irradiation, the content of MMP-1 in HDF cells was significantly increased (*p* < 0.001), and the content of type I collagen and type XVII collagen was significantly decreased (*p* < 0.01), indicating that UV irradiation could inhibit collagen synthesis and promote collagen degradation in HDF cells, and induce obvious cell photoaging damage. AKP treatment significantly reversed the damage caused by UV, improved the collagen content in photoaging HDF cells, and delayed the photoaging of cells.

### 2.7. Effect of AKP on Skin Appearance of Photoaging in Mice

**Figure 7:**
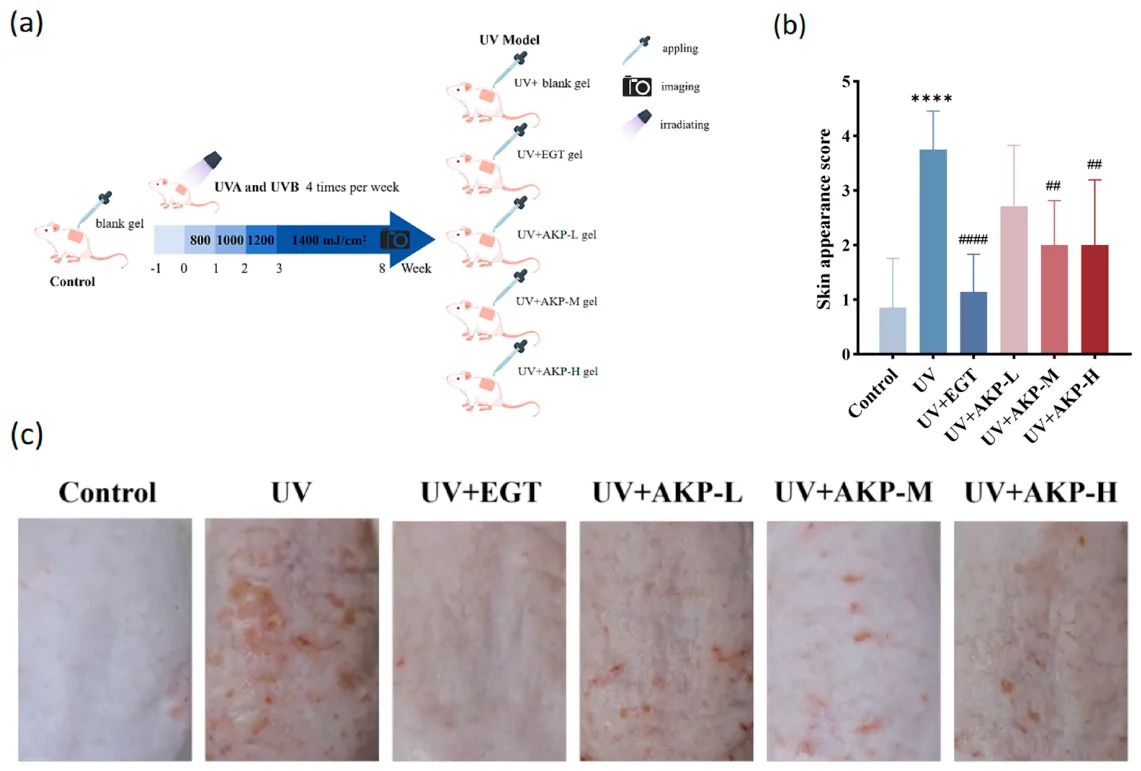
Effect of AKP on skin appearance of photoaging mice. (**a**) Ultraviolet irradiation scheme for animal experiments; (**b**) skin appearance score on the back of mice; (**c**) representative images of mouse back skin appearance. Results were expressed as mean ± SD (*n* = 8). **** *p* < 0.0001 compared with Control group; ^##^ *p* < 0.01, ^####^ *p* < 0.0001 compared with UV group.

After validating the protective effect of AKP in HDF cells, we further assessed its anti-photoaging activity in a UV-induced mouse model. The animal experiment plan and the ultraviolet irradiation plan are shown in Figure 7a. The mice were randomly divided into six groups: control group (Control), model group (UV), positive control ergothioneine group (UV+EGT), and AKP three dose groups (UV+AKP-L/M/H), which were carried out for 8 weeks. The irradiation intensity gradually increases, and the corresponding test substance is applied immediately after the irradiation. After the modeling, the back skin of the mice was photographed and scored. The results are shown in Figure 7b,c. The skin of the Control group was smooth and ruddy, full of elasticity, without wrinkles, erythema and skin damage. Compared with the Control group, the back skin of the mice in the UV group was dark and red, the skin was thickened, and the wrinkles were deepened. It was confirmed that UV irradiation could induce skin photoaging in mice, and the model was successfully established. After the intervention of EGT and AKP, the back skin of mice was improved to varying degrees, the skin darkness and redness subsided, the wrinkles decreased, and the epidermal thickening weakened, indicating that AKP treatment could help improve the skin photoaging phenotype and delay skin aging. At the same time, compared with the Control group, the skin appearance score of the UV group was significantly increased (*p* < 0.0001). After the intervention of EGT and AKP, the skin appearance scores of each group were reduced to varying degrees, indicating that AKP treatment could alleviate the skin damage caused by UV irradiation and improve the skin appearance as a whole.

### 2.8. The Regulatory Effect of AKP on the Pathological Morphology and Collagen Content of Photoaging Mice

**Figure 8:**
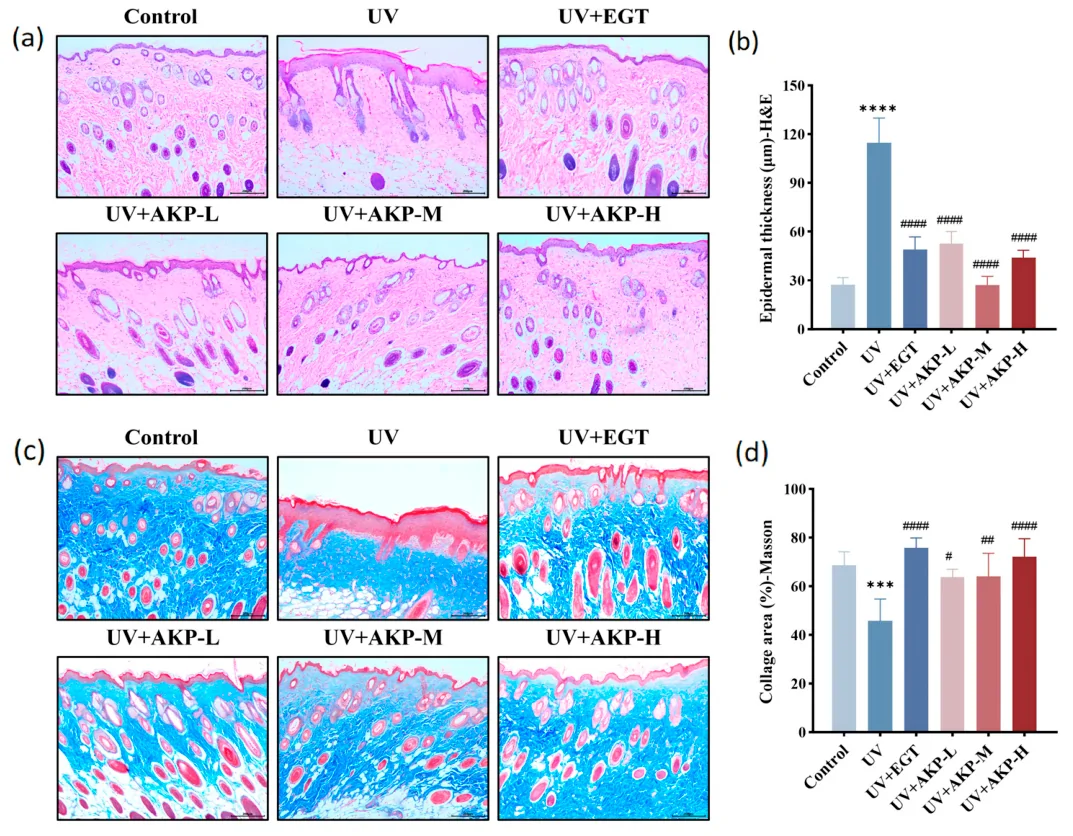
Effects of AKP on the pathological changes and collagen fiber area of skin tissue in photoaging mice. (**a**) H&E staining sections (scale = 200 μm); (**b**) epidermal thickness; (**c**) Masson staining sections (scale = 200 μm), blue represents collagen fibers; (**d**) collagen area. Results were expressed as mean ± SD (*n* = 8). *** *p* < 0.001, **** *p* < 0.0001 compared with Control group; ^#^ *p* < 0.05, ^##^ *p* < 0.01, ^####^ *p* < 0.0001 compared with UV group.

Hematoxylin and Eosin (H&E) staining was used to evaluate pathological changes. As shown in Figure 8a, the epidermis of the Control group was uniform, thin, and tightly connected, without keratinization or pathological alterations. The UV group exhibited significant epidermal hyperkeratosis, irregular thickening, and hyperplasia (*p* < 0.0001). AKP treatment significantly alleviated these abnormalities (*p* < 0.0001), with the medium-dose group showing the greatest reduction in epidermal thickness (Figure 8b). Masson staining (Figure 8c,d) revealed evenly distributed, wavy, dense collagen fibers in the Control group. In the UV group, dermal thickness was significantly reduced, collagen fibers became lighter and disordered, and numerous vacuolar defects appeared. AKP treatment significantly increased dermal thickness and collagen content (*p* < 0.05), indicating restoration of collagen architecture.

### 2.9. Effect of AKP on Antioxidant Capacity of Skin Tissue in Photoaging Mice

**Figure 9:**
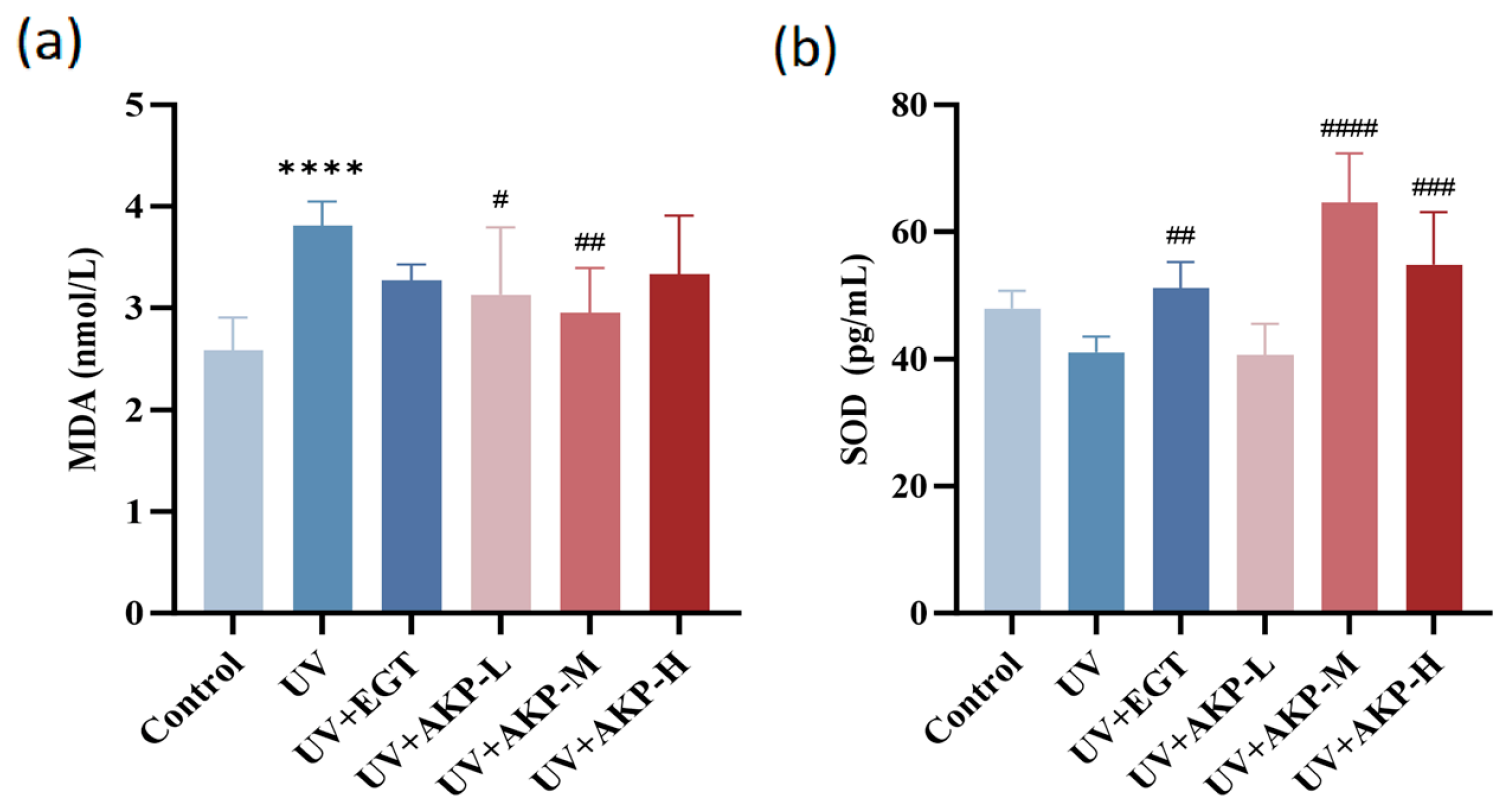
Effect of AKP on antioxidant capacity of skin tissue in photoaging mice. (**a**) MDA in skin tissue; (**b**) SOD in skin tissue. Results were expressed as mean ± SD (*n* = 8). **** *p* < 0.0001 compared with Control group; ^#^ *p* < 0.05, ^##^ *p* < 0.01, ^##*#*^ *p* < 0.001, ^####^ *p* < 0.0001 compared with UV group.

To determine whether AKP reduces oxidative damage in vivo, we measured MDA and SOD in skin tissues. As shown in Figure 9, UV irradiation significantly increased MDA (*p* < 0.0001) and decreased SOD activity compared with the Control group. AKP treatment reversed these changes, with the medium dose showing the most pronounced effect in reducing oxidative damage.

### 2.10. Proteomics Mining Key Proteins and Signaling Pathways of AKP in Improving Skin Photoaging

**Figure 10:**
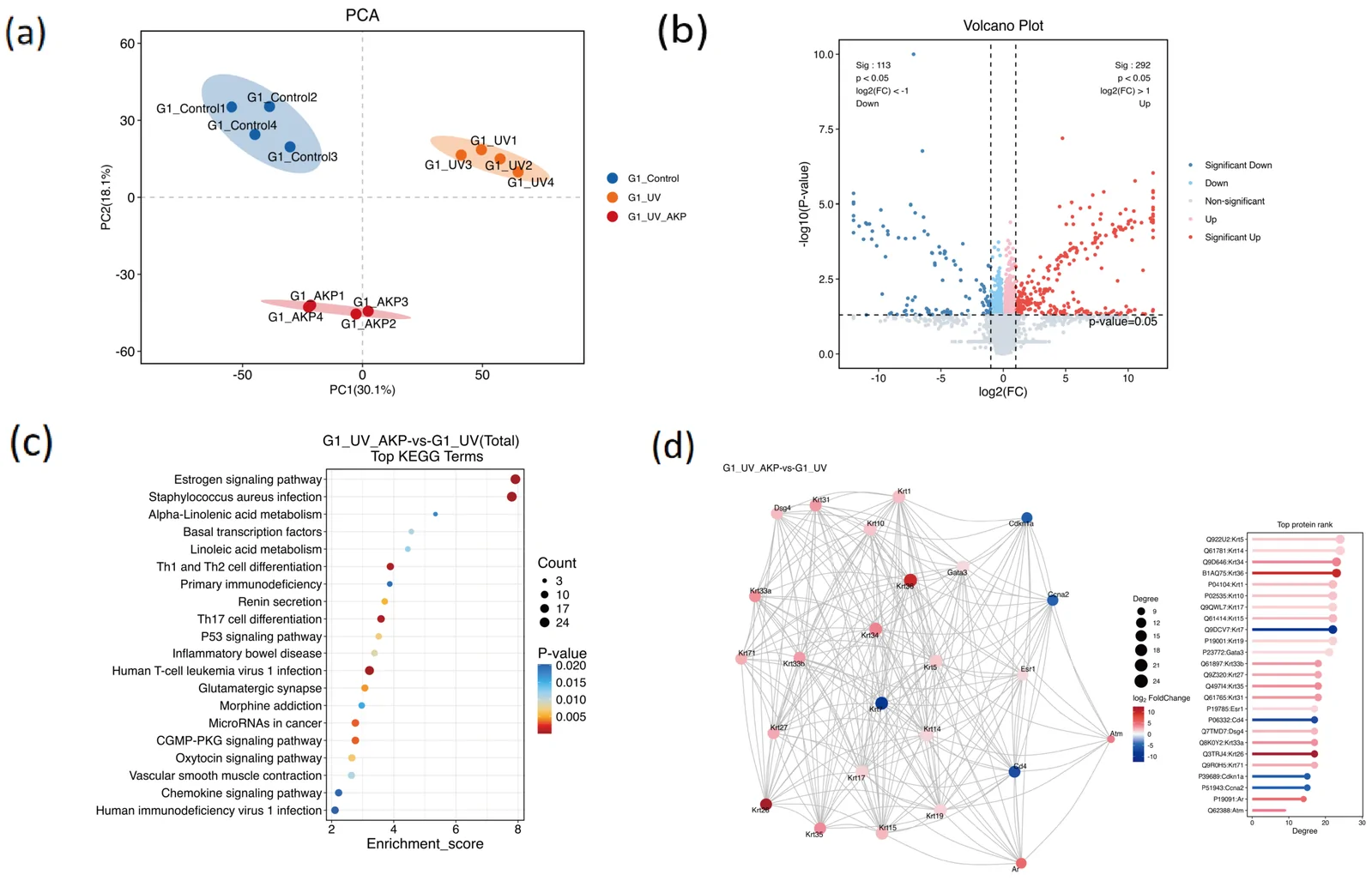
Proteomic identification of AKP-regulated proteins and pathways in UV-damaged mouse skin. (**a**) PCA principal component analysis; (**b**) differentially expressed protein volcano plot between UV_AKP group and UV group; (**c**) KEGG enrichment pathway diagram between UV_AKP group and UV group; in the figure, the horizontal axis is the enrichment fraction, and the vertical axis is the item/pathway information. The bubble size represents the number of proteins contained in the entry. The bubble color changes from blue to red. The more red the color is, the smaller the enrichment *p* value is, and the greater the significance. (**d**) Interaction network analysis of differentially expressed proteins between UV_AKP group and UV group. The left figure is the Top 25 connectivity protein interaction network diagram. The circle represents the differential protein/gene. Red represents up-regulation, blue represents down-regulation, and the size of the circle represents the degree of connectivity. The right figure is the Top 25 connectivity protein expression histogram; in the middle is the common legend of the two-part diagram.

Based on the above results, the medium-dose AKP group, which exhibited the best anti-photoaging effect, was selected for skin proteomic analysis to elucidate the underlying molecular mechanisms. Principal component analysis (PCA) (Figure 10a) showed tight clustering within groups and clear separation between groups, confirming good reproducibility. Differentially expressed proteins (DEPs) were screened using *p* < 0.05 and |fold change| ≥ 2 as criteria. As shown in Figure 10b, a total of 405 DEPs were identified between the UV_AKP and UV groups, of which 292 were significantly up-regulated and 113 down-regulated after AKP treatment.

KEGG pathway enrichment analysis of the DEPs (Figure 10c) revealed significant enrichment in the estrogen signaling pathway, Th1/Th2/Th17 cell differentiation, P53 signaling, cGMP-PKG, oxytocin, and chemokine signaling pathways, suggesting that AKP exerts regulatory effects through multiple pathways. Protein–protein interaction (PPI) network analysis (Figure 10d) identified the top 25 connectivity proteins, with the keratin family at the core. Notably, AKP intervention significantly up-regulated keratins Krt5, Krt14, Krt19, Krt1, and Krt10, while Krt7, which was markedly elevated in the UV group, was restored to near-normal levels after AKP treatment. Additionally, cell cycle regulators Cdkn1a and Ccna2 were significantly down-regulated, relieving cell cycle arrest and restoring normal proliferation. Crucially, AKP significantly up-regulated estrogen receptor Esr1 and androgen receptor Ar, which synergistically activated the estrogen signaling pathway, serving as a key molecular mechanism counteracting UV-induced photoaging. Collectively, these results indicate that AKP activates estrogen signaling via hormone receptor up-regulation, thereby reversing UV-induced damage through ROS scavenging, MMP-mediated collagen degradation inhibition, keratin network repair, and cell cycle normalization.

### 2.11. Verification of Differential Protein Expression

**Figure 11:**
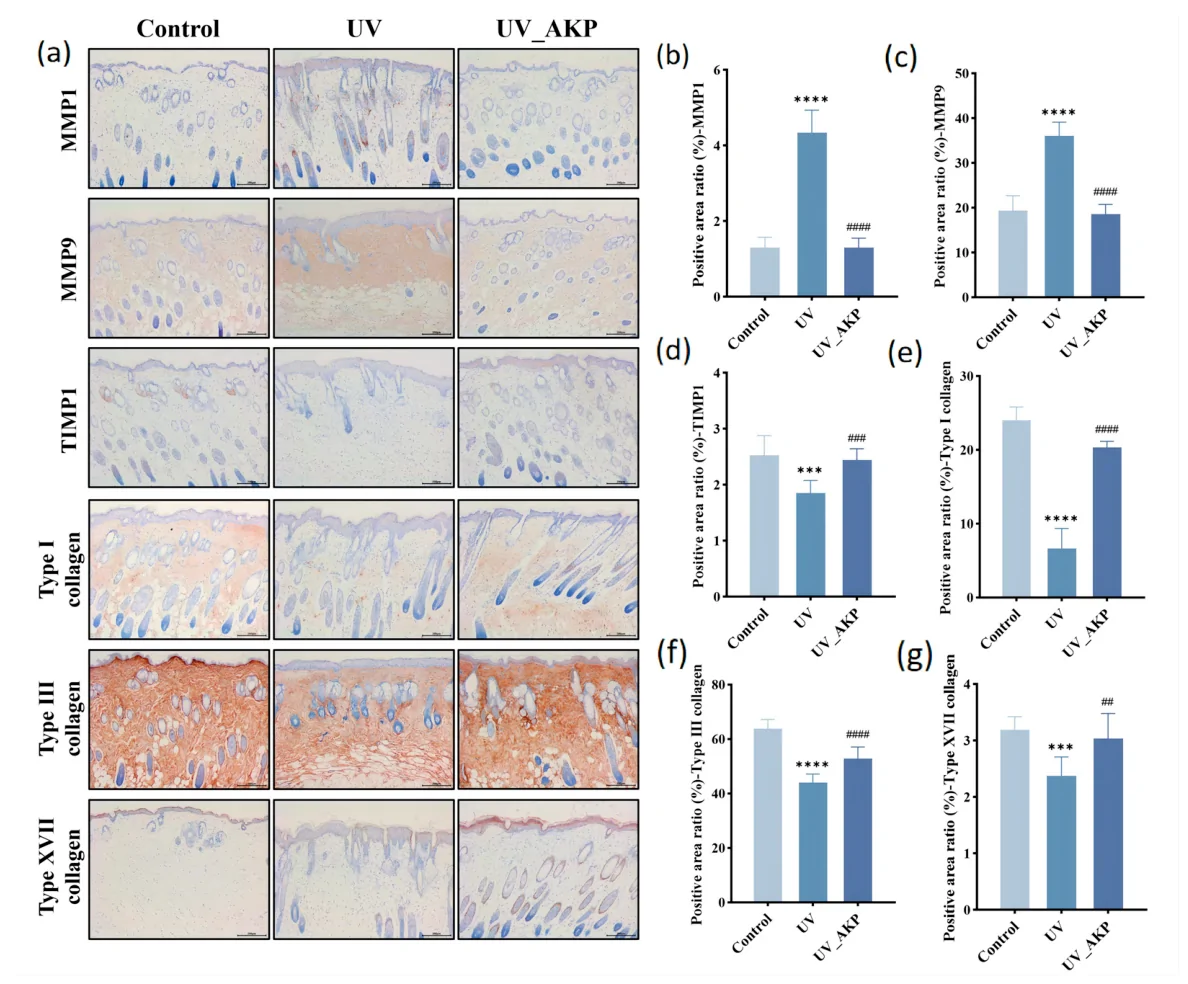
Effect of AKP on skin collagen expression in photoaging mice. (**a**) Immunohistochemical staining results (scale = 200 μm); (**b**–**g**) MMP1, MMP9, TIMP, type I collagen, type III collagen, and type XVII collagen positive area rate statistics. The brown part was the positive expression of protein; the results were expressed as mean ± SD. *** *p* < 0.001, **** *p* < 0.0001 compared with Control group; ^##^ *p* < 0.01, ^###^ *p* < 0.001, ^####^ *p* < 0.0001 compared with UV group.

Based on the pathway enrichment and PPI network results, we hypothesized that AKP repairs epidermal and dermal ECM imbalance primarily through estrogen signaling pathway activation and ECM homeostasis regulation. To validate this, we performed immunohistochemical staining for key matrix-related proteins. As shown in Figure 11, compared with the Control group, the UV group exhibited significantly increased expression of MMP-1 and MMP-9, along with decreased expression of their endogenous inhibitor TIMP1. Concomitantly, type I, III, and XVII collagens were markedly reduced, confirming UV-induced ECM degradation and basement membrane damage. AKP intervention significantly reversed these changes, suppressing excessive MMP expression, increasing TIMP1, and restoring collagen deposition. Importantly, the ratio of MMPs to TIMP1, a critical indicator of ECM homeostasis, was substantially elevated in the UV group and normalized by AKP treatment, underscoring its role in rebalancing matrix degradation and synthesis. These immunohistochemical findings are highly consistent with the proteomic data, directly confirming that AKP can achieve photoaging repair by activating the estrogen signaling pathway and maintaining ECM homeostasis.

## 3. Discussion

Skin photoaging is a premature aging caused by long-term exposure to excessive ultraviolet radiation, which is closely related to the environment and living habits [27,28]. Current clinical interventions face limitations in achieving safe, long-term efficacy, driving interest in natural, efficient, and readily absorbable anti-photoaging agents from marine resources [29]. Although Antarctic krill peptide exhibit diverse bioactivities, their role in skin photoprotection remains underexplored. In this study, we demonstrated that AKP, a low-molecular-weight peptide fraction (Mw ~331 Da, >82% <500 Da) prepared by enzymatic hydrolysis and membrane separation, exerts potent anti-photoaging effects through a novel mechanism involving estrogen signaling pathway activation and ECM homeostasis regulation, as revealed by integrated proteomic and immunohistochemical analyses.

In order to obtain the Antarctic krill peptides AKP that meets the experimental requirements, the extraction and preparation of raw materials and the evaluation of transdermal absorption in vitro were first carried out. The physicochemical properties of bioactive peptides have a decisive influence on their transdermal efficacy. Usually, the lower the molecular weight, the stronger the skin permeability and biological activity [30]. Antarctic krill crude hydrolysates usually exhibit a wide molecular weight distribution, resulting in poor dispersion and targeting of active ingredients [31]. Our membrane-based fractionation strategy yielded an AKP fraction with a narrow distribution and exceptional in vitro skin permeability (~91% cumulative permeation at 24 h). Notably, Antarctic krill is also rich in lipophilic constituents, such as astaxanthin, which may synergistically enhance membrane partitioning and interaction with cutaneous receptors, partly accounting for the favorable transdermal profile observed. These characteristics establish AKP as a viable candidate for topical photoprotection and provide a standardized preparation protocol applicable to other marine plankton-derived peptides.

Human skin fibroblasts (HDF) are the core functional cells that synthesize collagen and maintain skin elasticity in the dermis. They are also the main target cells of UV radiation damage and are often used for skin photoaging research [13,32]. In our UV-irradiated HDF model, AKP treatment effectively restored antioxidant enzyme activity, reduced the accumulation of ROS and MDA, and reversed MMP-1 up-regulation while promoting type I and XVII collagen secretion. These findings are consistent with the effect of marine bioactive peptides such as salmon skin collagen peptides on alleviating UV-induced oxidative stress through endogenous antioxidant pathways [33]. Collagen fibers and elastic fibers in the dermis of the skin are arranged in an orderly and dense manner, and cross-linked to build a network support structure to maintain the mechanical toughness and elasticity of the skin [34]. Skin photoaging will aggravate the loss of collagen, resulting in weakened dermal elasticity and loose wrinkles on the epidermis [35]. Among them, type I collagen is the main component of skin collagen, and XVII collagen is a transmembrane collagen existing in the basal layer of the epidermis. Both of them can anchor the epidermis and dermis, and are the key factors to maintain the stability of skin structure [36]. Consistent with the results of Hu et al. [37], UV irradiation can lead to a decrease in the secretion of type I and XVII collagen in HDF cells, and up-regulate the expression of MMP-1 to accelerate collagen degradation, causing ECM remodeling imbalance, and ultimately leading to skin photoaging. AKP treatment can improve this disorder by down-regulating the expression of MMP-1 and promoting collagen synthesis, maintaining the stability of skin structure, and providing in vitro data support for subsequent animal experiments.

In vivo, the chronic UV-induced mouse photoaging model more faithfully recapitulates the complex histological and macroscopic features of human skin aging, including epidermal hyperplasia, dermal ECM degradation, and wrinkle formation [38]. Therefore, a mouse model of chronic skin photoaging was constructed by UV induction, and an eight-week irradiation experiment was carried out to simulate the exogenous skin aging process caused by long-term ultraviolet irradiation. Skin surface wrinkles and erythema are often used to assess the severity of photoaging [39]. The back skin of mice in the UV group was dark red and swollen, the erythema increased, and the wrinkles deepened. The erythema wrinkles were reduced after AKP treatment, which could alleviate the effect. Epidermal hyperplasia is a histological feature of photoaging skin, which can reflect the degree of skin damage induced by ultraviolet radiation [40]. AKP treatment can improve the abnormal thickening of the epidermis of UV mice, and the pathological symptoms are also alleviated. Masson trichrome staining is usually used to determine the distribution of skin collagen, consistent with the results of Chen et al. [41], AKP has a protective effect on collagen fiber damage and skin collagen content reduction caused by UV irradiation. In addition, at the animal level, corresponding to the cell level, AKP can also improve the activity of antioxidant enzymes and exert antioxidant capacity, which fully proves that AKP still has excellent anti-photoaging effect in a complex skin tissue environment.

To unravel the underlying molecular mechanisms, we performed label-free proteomic profiling of skin tissues from UV-exposed and AKP-treated mice. KEGG enrichment analysis revealed that differentially expressed proteins were significantly enriched in the estrogen signaling pathway, alongside inflammation-, senescence-, and metabolism-related pathways. The estrogen signaling axis is known to regulate skin structural protein synthesis, inhibit collagen degradation, and counteract oxidative and inflammatory damage [42]. Intriguingly, AKP intervention significantly up-regulated estrogen receptor Esr1 and androgen receptor Ar, suggesting that AKP may function as a selective estrogen receptor modulator-like agent. PPI network analysis further identified keratins (Krt5, Krt14, Krt19, Krt1, Krt10) as core connectivity hubs, with aberrant UV-induced expression effectively normalized by AKP. Concurrently, cell cycle regulators Cdkn1a and Ccna2 were down-regulated, relieving UV-induced cell cycle arrest and restoring proliferative capacity. Critically, immunohistochemical validation confirmed that AKP suppressed UV-induced overexpression of MMP-1 and MMP-9, elevated their endogenous inhibitor TIMP1, and restored collagens I, III, and XVII. Importantly, the MMPs/TIMP1 ratio, a key determinant of ECM homeostasis, was markedly elevated in UV-exposed skin and substantially normalized by AKP, underscoring its role in rebalancing matrix degradation and synthesis. Collectively, these findings delineate a comprehensive regulatory cascade in which AKP up-regulates Esr1 and Ar, leading to activation of the estrogen signaling pathway. This activation subsequently suppresses AP-1/NF-κB-driven MMP transcription, thereby inhibiting collagenolysis and promoting collagen deposition, which ultimately restores ECM homeostasis and basement membrane repair. This pathway-centric mechanism distinguishes AKP from conventional antioxidant-based anti-photoaging agents and positions it as a promising multi-target therapeutic.

Compared with other marine-derived anti-photoaging peptides, such as Pacific cod gelatin polypeptides (which act via TGF-β/Smad signaling) [17] and *Pinctada martensii* peptides (which reduce MMPs through direct enzyme inhibition) [18], AKP exhibits a unique advantage in directly targeting hormone receptors (Esr1/Ar), thereby orchestrating a broader transcriptional reprogramming of ECM-related genes. This receptor-mediated mechanism may also explain the relatively low effective concentration (0.4 mg/mL) observed in our cellular assays. Furthermore, the presence of lipophilic constituents in Antarctic krill may facilitate not only transdermal delivery but also receptor-level interactions, offering a dual advantage over purified peptide fractions from other sources.

There are still some limitations in this study to be further explored. First, AKP is a complex oligopeptide mixture rather than a single characterized peptide; future work will focus on isolating and identifying the specific sequence(s) responsible for Esr1/Ar binding. Second, while topical application demonstrated efficacy, the actual biodistribution and concentration of AKP in target skin layers were not directly quantified. Third, although immunohistochemistry confirmed changes in estrogen pathway components, direct genetic or pharmacological validation (e.g., using Esr1 knockout or selective antagonists) is needed to establish causality. These investigations are currently underway in our laboratory and will provide more direct molecular evidence for the proposed mechanism.

## 4. Materials and Methods

### 4.1. Materials and Reagents

Defatted Antarctic krill powder was obtained from Shandong Luhua Marine Biotechnology Co., Ltd. (Jinan, China). Flavor protease was obtained from Beijing Solarbio (Beijing, China). Human dermal fibroblasts (HDF) were obtained from Suzhou Beina Chuanglian (Suzhou, China). ELISA kits for MMP-1 (ZX-03227H1), type I collagen (ZX-03329H1), and type XVII collagen (ZX-6419H1) were obtained from Qingdao Zhongxi (Qingdao, China). MDA and SOD kits were obtained from Beijing Solebao (Beijing, China). Antibodies Col I, Col III, Col XVII, MMP-1, MMP-9 and TIMP-1 were obtained from BioLegend (San Diego, CA, USA). All reagents were of analytical grade.

### 4.2. Preparation of AKP

AKP was prepared by enzymatic hydrolysis and membrane fractionation. Defatted Antarctic krill powder was dissolved in distilled water, adjusted to neutral pH, and hydrolyzed with flavor protease (5600 U/g) at 50.7 °C for 6.5 h. The enzyme was inactivated at 90–100 °C for 10 min, and the hydrolysate was centrifuged, decolorized, and filtered. The supernatant was sequentially fractionated through 500 Da ultrafiltration and 200 Da nanofiltration membranes. The 200–500 Da fraction was collected and lyophilized to obtain AKP powder. Molecular weight distribution was determined by HPLC-SEC (Agilent Technology Co., Ltd., Beijing, China) (TSKgel G2000 SWXL column, 0.1 M phosphate buffer pH 6.7 with 0.1 M sodium sulfate, 1.0 mL/min, 220 nm) using peptide standards.

### 4.3. Liquid Chromatography-Tandem Mass Spectrometry

The AKP samples were hydrolyzed for protein quantification, and then the appropriate amount of protein samples were taken. The 10 kDa ultrafiltration tube was used for ultrafiltration, and the flow through liquid was collected. After desalination by C18 desalination column, freeze-dried for use. The peptides were dissolved in 0.1% formic acid aqueous solution and analyzed by Orbitrap Fusion Lumos mass spectrometer with Vanquish Neo UHPLC in series (Thermo Fisher Scientific Co., Ltd., Shanghai, China). Specific chromatographic conditions: chromatographic column: C18 chromatographic column (20 cm × 75 μm i.d., 1.9 μm); column temperature 40 °C; mobile phase A: 0.1% formic acid; mobile phase B: 0.1% formic acid, 80% acetonitrile; the flow rate was 300 nL/min. Mass spectrometry parameters: scan range: 200–1550 *m*/*z*; resolution: 120,000; normalized automatic gain control (AGC) target value: 300%; the maximum injection time: 20 ms. secondary mass spectrometry parameters: resolution: 15,000; AGC target value: 200%; the maximum injection time: 22 ms; collision energy: 32%; the dynamic exclusion time was 35 s. The original mass spectrometry files were retrieved from the uniprot-Euphausiidae database (Antarctic krill, version 2025,42647 entries) using PEAKS Studio version 13 software.

### 4.4. In Vitro Transdermal Absorption

Full-thickness dorsal skin from Kunming mice was mounted on Franz diffusion cells (effective area 2.54 cm^2^) with the stratum corneum intact. Donor compartment: 3 mL AKP (10 mg/mL in saline); receptor: 6 mL saline at 37 °C with stirring (600 r/min). At 1, 2, 4, 6, 8, 10, and 24 h, 0.5 mL aliquots were withdrawn and replaced with fresh saline. AKP concentration in receptor fluid was measured by BCA. Cumulative permeation (*Q_n_*) was calculated as:

$$Q_{n}\;=\;\frac{V\rho_{n}+V_{0}\sum_{i=1}^{n-1}\rho_{i}}{A}$$

In the formula, *Q_n_* is the cumulative permeation amount per unit area of the nth sampling point, mg/cm^2^; *V* is receptor volume (6 mL), *V*_0_ is sampling volume (0.5 mL), and *A* is diffusion area.

### 4.5. Cell Experiments

#### 4.5.1. Cell Culture

HDF cells were cultured in DMEM with 10% FBS and 1% penicillin/streptomycin at 37 °C in 5% CO_2_. Cells at passages 3–8 were used.

#### 4.5.2. Cytotoxicity and UV Dose Optimization

Cells were seeded in 96-well plates (1 × 10^4^/well). For cytotoxicity, cells were treated with AKP (0.2–2.0 mg/mL) for 24 h, and viability was assessed by MTT. For UV dose selection, cells in PBS were exposed to 100–800 mJ/cm^2^ UV (UVA/UVB) and cultured for 24 h before MTT assay. The dose yielding ~60% viability (600 mJ/cm^2^) was chosen for subsequent experiments.

#### 4.5.3. Protective Effect and Biochemical Assays

UV-irradiated cells were treated with AKP (0.2–2.0 mg/mL) for 24 h, and viability was measured by MTT. Based on results, 0.4 mg/mL AKP was selected for mechanistic assays. Culture supernatants were collected for ELISA (MMP-1, type I collagen, type XVII collagen). Cells were lysed by ultrasonication, and SOD activity and MDA content were measured using commercial kits per manufacturer protocols.

#### 4.5.4. Determination of Intracellular ROS Levels

Cell culture according to 4.5.3. After treatment, the cells were washed with serum-free medium, and then the DCFH-DA probe was diluted with serum-free medium (1:1000); 1 mL probe diluent was added to each well in the dark. After incubation at 37 °C for 30 min, 1 mL PBS was added to each well after washing the cells. The cells were observed and photographed under a fluorescence inverted microscope (Shanghai Changfang Optical Instrument Co., Ltd., Shanghai, China) (excitation wavelength 488 nm, emission wavelength 525 nm). The fluorescence intensity of cells in each group was analyzed by Image J (1.54d) software.

### 4.6. Animal Experiments

#### 4.6.1. Animals and Ethics

SPF male Kunming mice (4–5 weeks, 20–25 g) were from Jinan Pengyue (Jinan, China) (License No.: SCXK (Shandong) 2022-0006). All procedures were approved by the Animal Ethics Committee of Ocean University of China (Approval No.: SPXY2023090710). Mice were housed under standard conditions (22 ± 2 °C, 50 ± 5% humidity, 12 h light/dark) with food and water ad libitum.

#### 4.6.2. UV Irradiation and Treatment

Mice were randomly divided into 6 groups (*n* = 8): Control, UV, UV+EGT (10 mg/mL, positive control), UV+AKP-L (10 mg/mL), UV+AKP-M (50 mg/mL), and UV+AKP-H (100 mg/mL). Doses were based on preliminary experiments. Dorsal skin was exposed to UV (UVA/UVB) four times weekly for 8 weeks, with gradually increasing doses (1.0 J/cm^2^ UVA + 0.05 J/cm^2^ UVB in week 1 to 4.0 + 0.2 J/cm^2^ by week 8). Immediately after each irradiation, 0.5 mL of corresponding gel was topically applied. On day 57, skin was photographed, and mice were euthanized. Dorsal skin was collected and stored at −80 °C.

#### 4.6.3. Skin Appearance Scoring

Skin photoaging severity was scored by blinded observers from 0 (normal) to 5 (severe) based on wrinkles, erythema, and thickening, according to published criteria [40].

#### 4.6.4. Histological Staining

Skin sections (4 μm) were stained with H&E for epidermal thickness measurement and Masson’s trichrome for collagen fiber visualization. The skin tissue of mice was taken, cleaned and fixed in 4% paraformaldehyde solution for 48 h to prepare paraffin sections of skin tissue. H&E stained sections were prepared by hematoxylin–eosin staining; the sections of Masson staining need to be dewaxed and rehydrated first, and then mordant stained overnight at room temperature with 2.5% potassium dichromate mordant. After washing, hematoxylin staining was performed for 6 min, 1% hydrochloric acid ethanol was used for rapid differentiation for several seconds, saturated lithium carbonate was returned to blue for 1 min, and then stained with Lichun Red dye solution for 6 min. After washing, the sections were differentiated by 1% phosphomolybdic acid for 5 min, aniline blue for 2 min, and 1% glacial acetic acid for 1 min. The differentiation steps were repeated several times. Finally, the sections were prepared by dehydration, transparency and sealing. Images were captured by light microscopy (Olympus Corporation, Tokyo, Japan), and quantitative analysis (epidermal thickness, collagen area) was performed using ImageJ (1.54d).

#### 4.6.5. Oxidative Stress Markers

Skin tissue (100 mg) was homogenized in PBS (1:9, *w*/*v*). After centrifugation (10,000 r/min, 15 min, 4 °C), supernatant MDA and SOD were measured using commercial kits.

### 4.7. Proteomic Analysis

Skin tissues from UV and UV+AKP-M groups (*n* = 4 each) were subjected to label-free quantitative proteomics. Proteins were extracted, reduced (10 mM DTT, 55 °C, 30 min), alkylated (20 mM iodoacetamide, dark, 20 min), and digested sequentially with Lys-C and trypsin. Peptides were desalted on SOLA™ SPE plates. LC-MS/MS was performed on an Orbitrap Fusion Lumos with Vanquish Neo UPLC HSS T3 column (Thermo Fisher Scientific, Waltham, MA, USA). Mobile phase A: 0.1% formic acid in water; B: acetonitrile. Gradients: 5–25% B (40 min), 25–50% B (20 min), 50–90% B (5 min). MS1 resolution 180,000; scan range 150–2000 *m*/*z*. Raw data were processed with MaxQuant (v2.0.3.0) against the UniProt mouse database (UP000000589). Differentially expressed proteins (|FC| ≥ 2, *p* < 0.05) were subjected to KEGG enrichment and STRING-based PPI analysis.

### 4.8. Immunohistochemistry

Skin sections (4 μm) underwent antigen retrieval (citrate buffer pH 6.0, boiling 15 min), peroxidase blocking (3% H_2_O_2_, 25 min), and BSA blocking (30 min). Sections were incubated overnight at 4 °C with primary antibodies (Col I, Col III, Col XVII, MMP-1, MMP-9, TIMP1), followed by HRP-conjugated secondary antibodies (50 min). Immunoreactivity was visualized with DAB, counterstained with hematoxylin, and imaged. Positive areas were quantified using ImageJ (1.54d).

### 4.9. Statistical Analysis

Data are mean ± SD. Comparisons among multiple groups used one-way ANOVA with LSD post hoc test (GraphPad Prism 10.0). *p* < 0.05 was considered significant. Significance levels are indicated as *p* < 0.05, <0.01, <0.001, and <0.0001.

## 5. Conclusions

In summary, we successfully prepared a low-molecular-weight Antarctic krill peptide mixture (AKP, Mw ~331 Da) via enzymatic hydrolysis and membrane fractionation, which exhibited excellent transdermal permeability. Both in vitro and in vivo experiments demonstrated that AKP effectively alleviates UV-induced skin photoaging by reducing oxidative stress, inhibiting MMP-mediated collagen degradation, and promoting the synthesis of type I, III, and XVII collagens. Mechanistically, integrated proteomic and immunohistochemical analyses revealed that AKP activates the estrogen signaling pathway through up-regulation of Esr1 and Ar, which subsequently restores extracellular matrix homeostasis and repairs basement membrane integrity. This study is the first to establish a mechanistic link between Antarctic krill-derived peptides and estrogen-mediated photoprotection, filling a notable gap in the field. Collectively, our findings position AKP as a promising natural candidate for topical anti-photoaging applications and provide a theoretical foundation for the development of marine-based functional ingredients in next-generation skincare products.

## Figures and Tables

**Table 1:** Identification results of the first 15 peptides in AKP abundance expression.

Peptide	Mass (Da)	Length	*m*/*z*	Retention Time (min)	Expression Abundance
IPP	325.2001	3	326.2071	6.0123	92,600,000
API	299.1845	3	300.1912	4.9249	90,000,000
APL	299.1845	3	300.1912	4.9249	90,000,000
TPI	329.1951	3	330.2018	6.5680	81,100,000
TPL	329.1951	3	330.2018	6.5680	81,100,000
IPL	341.2314	3	342.2385	31.6482	75,300,000
DWH	456.1757	3	457.1808	0.1922	67,900,000
LPT	329.1951	3	330.2015	5.8164	63,100,000
VPGV	370.2216	4	371.2282	0.3936	63,000,000
GWH	398.1702	3	399.1752	0.3391	48,900,000
RSW	447.2230	3	448.2278	5.7225	47,600,000
VPI	327.2158	3	328.2226	12.4787	47,600,000
VPL	327.2158	3	328.2226	12.4787	37,200,000
IPV	327.2158	3	286.1756	2.6743	35,700,000
IPG	285.1689	3	286.1761	0.0086	48,900,000

## Data Availability

Data generated and analyzed in this study can be obtained from the corresponding authors upon reasonable request.

## Supplementary Materials

The following supporting information can be downloaded at https://www.mdpi.com/article/10.3390/md24100342/s1, Figure S1: HPLC-SEC chromatograms of standard curve and AKP.
