# Supplementary material for: A Preliminary Study on the Effect and Mechanism of Antarctic Krill Peptides AKP on Alleviating UV-Induced Skin Photoaging by Regulating Estrogen Signaling Pathway
Source: Mar Drugs. 2026 Sep 29;24(10):342. doi: 10.3390/md24100342 (PMC13706157; doi:10.3390/md24100342)
Supplement: Supplementary file 1 [file marinedrugs-24-00342-s001.zip › marinedrugs-4551806-supplementary.pdf]

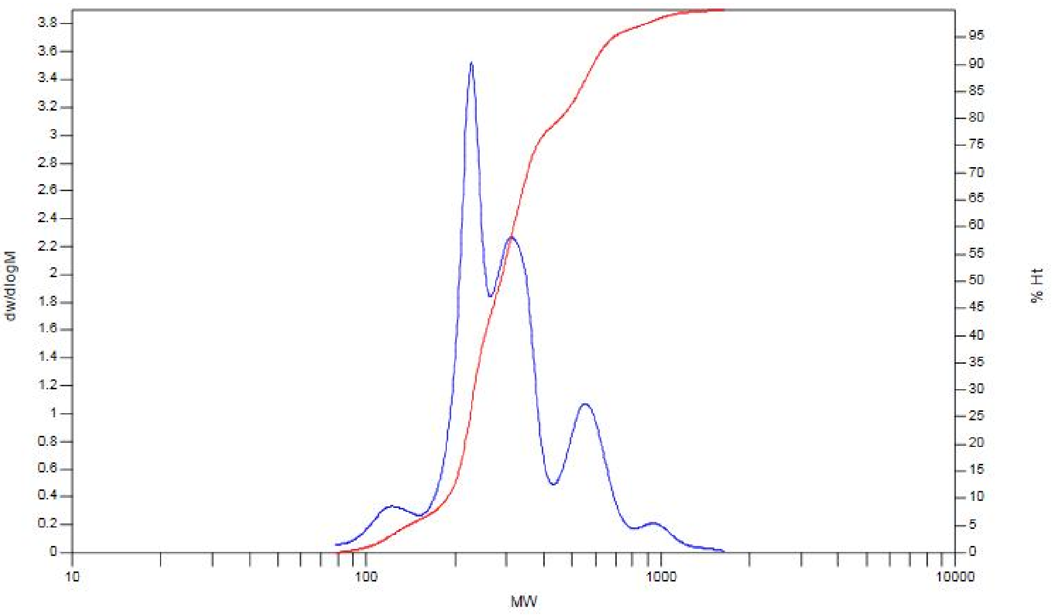

Figure S1. HPLC-SEC chromatograms of standard curve and AKP.
